# Supplementary material for: AnnoSpat annotates cell types and quantifies cellular arrangements from spatial proteomics
Source: Nat Commun. 2024 May 3;15:3744. doi: 10.1038/s41467-024-47334-0 (PMC11068798; doi:10.1038/s41467-024-47334-0)
Supplement: Supplementary file 1 — Supplemental Information [file 41467_2024_47334_MOESM1_ESM.pdf]

Supplementary Information: AnnoSpat annotates cell types and quantifies cellular arrangements from spatial proteomics

Aanchal Mongia[1,2], Fatema Tuz Zohora[3], Noah G. Burget[1,2], Yeqiao Zhou[1,2], Diane C. Saunders[4], Yue J. Wang[5], Marcela Brissova[4], Alvin C. Powers[4,6,7], Klaus H. Kaestner[2,5,8], Golnaz Vahedi[2,5,8], Ali Naji[8,9], Gregory W. Schwartz[3,10,11,*], and Robert B. Faryabi[1,2,*]

[1]Department of Pathology and Laboratory Medicine, University of Pennsylvania Perelman School of Medicine, Philadelphia, PA, USA
[2]Epigenetics Institute, University of Pennsylvania Perelman School of Medicine, Philadelphia, PA, USA
[3]Princess Margaret Cancer Centre, University Health Network, Toronto, ON, Canada
[4]Department of Medicine, Vanderbilt University Medical Center, Nashville, TN, USA
[5]Department of Genetics, University of Pennsylvania Perelman School of Medicine, Philadelphia, PA, USA
[6]Department of Molecular Physiology and Biophysics, Vanderbilt University, Nashville, TN, USA
[7]VA Tennessee Valley Healthcare System, Nashville, TN, USA
[8]Institute for Diabetes, Obesity and Metabolism, University of Pennsylvania Perelman School of Medicine, Philadelphia, PA, USA
[9]Department of Surgery, University of Pennsylvania Perelman School of Medicine, Philadelphia, PA, USA
[10]Department of Medical Biophysics, University of Toronto, Toronto, Canada
[11]Vector Institute, University of Toronto, Toronto, Canada
[*]Co-corresponding authors

Supplementary Figure 1

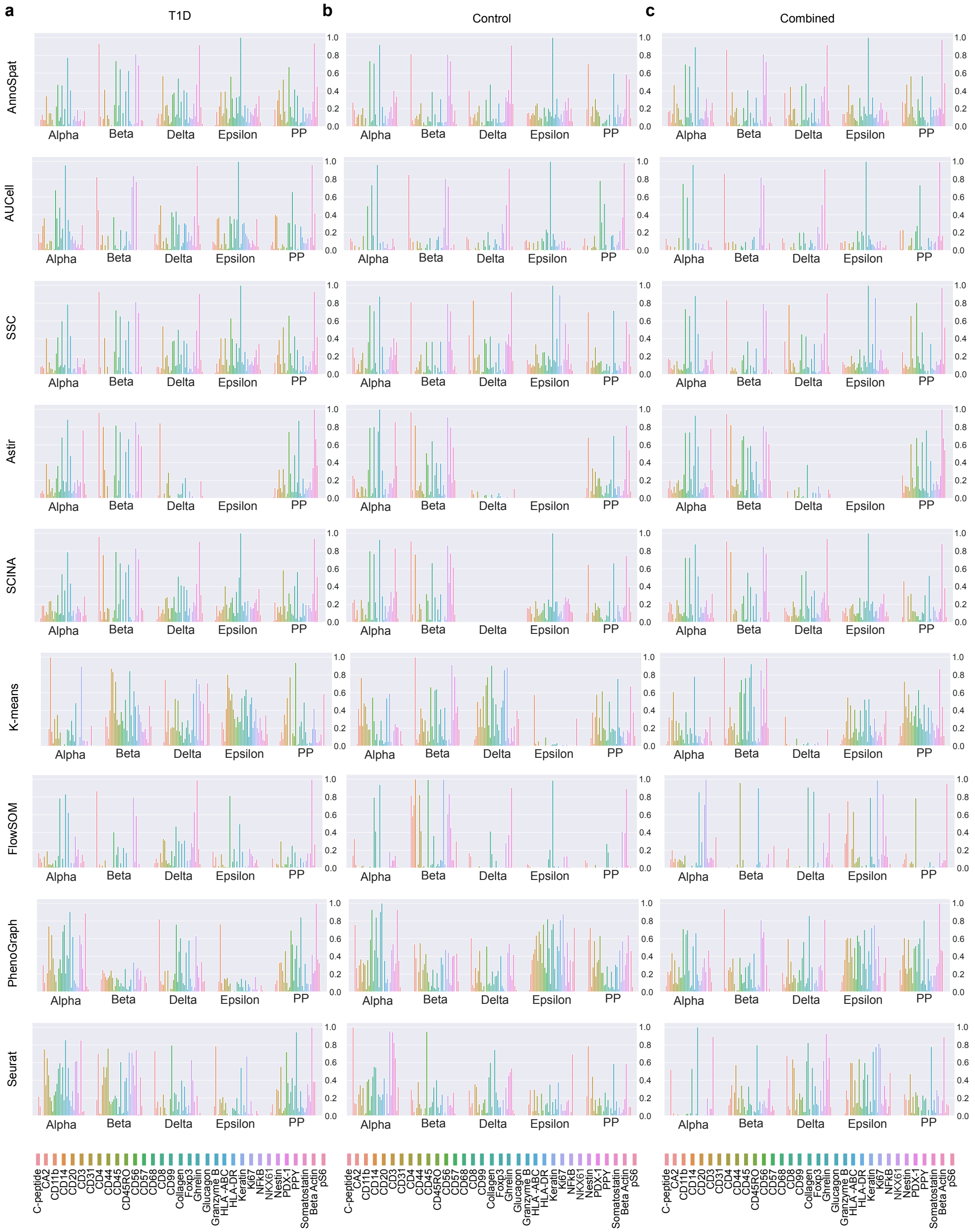

Supplementary Figure 1: Normalized average expression of endocrine cell canonical markers in alpha, beta, delta, epsilon, and PP-labels cells. **a** From top to bottom: bar plots showing normalized average expression of all the 33 HPAP IMC-measured proteins for cells annotated as alpha, beta, delta, epsilon, and PP by AnnoSpat, AUCell, our semi-supervised clustering (SSC), Astir, SCINA, K-means, FlowSOM, PhenoGraph, and Seurat from T1D pancreas IMC data ($n =$ 374,397 measured cells). **b** Similar to (a) from non-diabetic (control) pancreas IMC data ($n =$ 795,604 measured cells). **c** Similar to (a) from combined T1D and control pancreas IMC data ($n =$ 1,170,001 measured cells).

Supplementary Figure 2

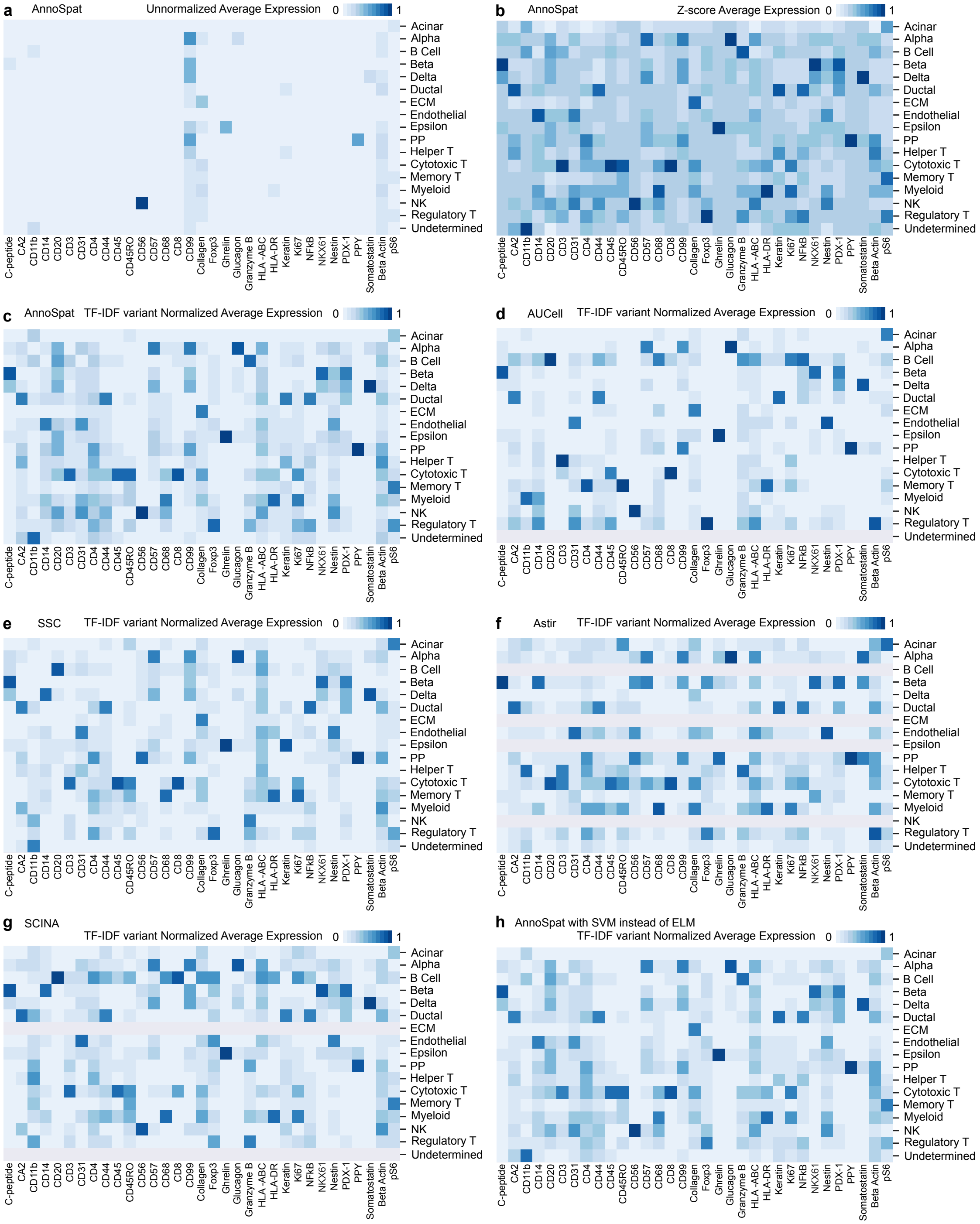

Supplementary Figure 2: Heatmaps showing average unnormalized **a**, protein-wise z-score normalized **b**, and a variant of TF-IDF normalized **c-h** expression levels of all the 33 HPAP IMC-measured proteins across AnnoSpat (c), AUCell (d), our SSC (e), Astir (f), SCINA (g), and AnnoSpat with SVM instead of ELM (h) annotated cell types from combined T1D and control pancreas IMC data ($n =$ 1,170,001 measured cells). Heatmaps comparison indicates the benefit of a variant of TF-IDF for normalization in visualizing continuous protein expression readouts. Note: TF-IDF variant normalization is only used for data visualization and not cell-type annotation.

Supplementary Figure 3

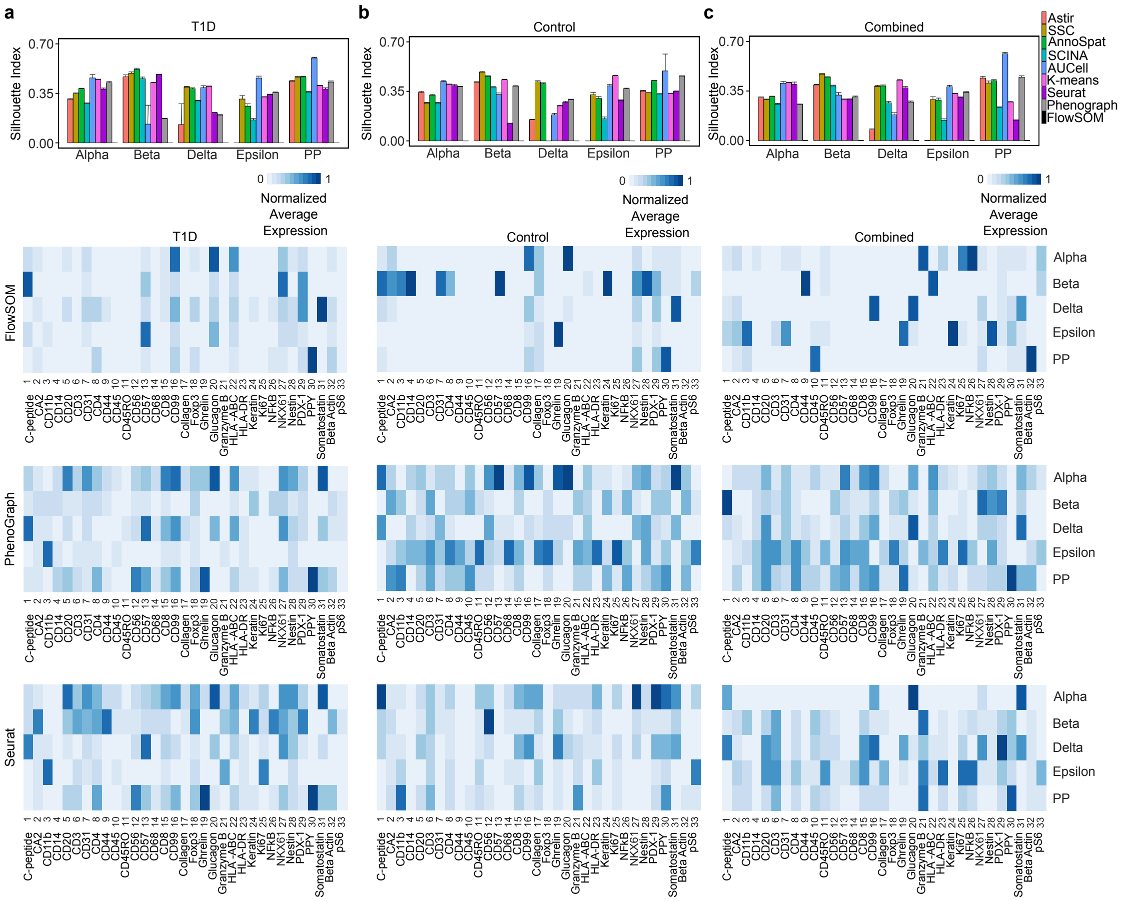

Supplementary Figure 3: Comparative analysis of AnnoSpat cell-type annotation from IMC data with clustering-based cell-type annotation methods. **a** From top to bottom: bar plots with error bars showing average and standard deviation Silhouette Index (SI), heatmaps showing normalized average expression of all the 33 HPAP IMC-measured proteins for cells annotated as alpha, beta, delta, epsilon, and PP by FlowSOM, PhenoGraph, and Seurat from T1D pancreas IMC data ($n = 374{,}397$ measured cells). **b** Similar to (a) from non-diabetic (control) pancreas IMC data ($n = 795{,}604$ measured cells). **c** Similar to (a) from combined T1D and control pancreas IMC data ($n = 1{,}170{,}001$ measured cells). $m = 20$ sets of $n = 50{,}000$ randomly selected cells are used for evaluation using SI in each bar plot in top panel of a-c.

Supplementary Figure 4

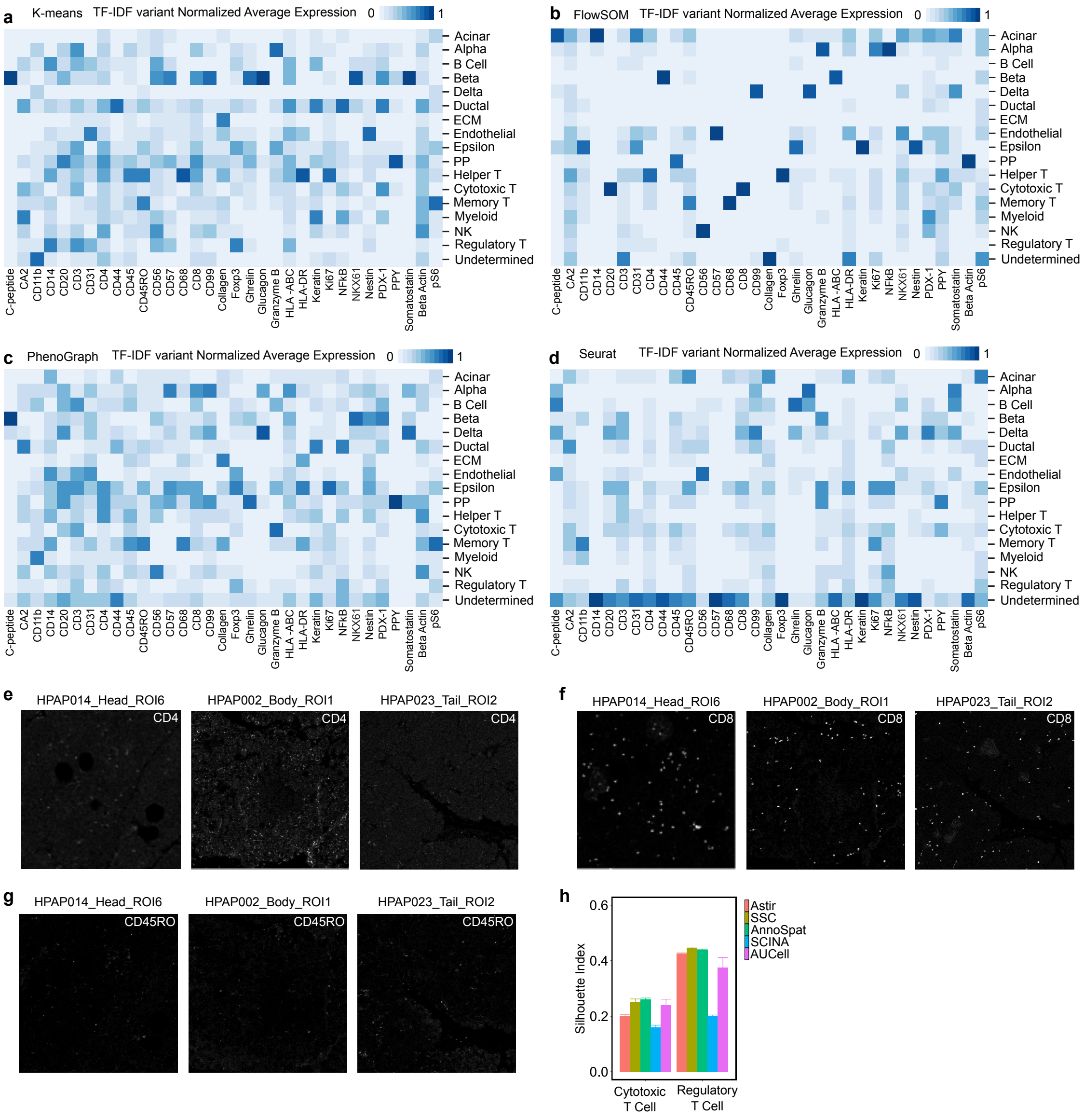

Supplementary Figure 4: Visualization of variability in staining quality of antibodies used in the HPAP IMC panel. **a-d** normalized average expression of all the 33 HPAP IMC-measured proteins for K-means (a), FlowSOM (b), PhenoGraph (c), and Seurat (d) annotated cell types from combined T1D and control pancreas IMC data (n = 1,170,001 measured cells). **e-g** Randomly selected IMC images of ROIs from pancreas head, body, tail comparing CD4 (e), CD8 (f), and CD45RO (g) staining quality showing higher quality of CD8 compared to CD4 and CD45RO staining. **h** Bar plots with error bars showing average and standard deviation of Silhouette Index (SI) values for cells annotated as $CD8^+$ T cells and regulatory T cells by AnnoSpat semi-supervised clustering (SSC), Astir, SCINA, and AUCell from combined T1D and control pancreas IMC data (n = 1,170,001 measured cells). m = 20 sets of n = 50,000 randomly selected cells are used for evaluation using SI in each bar plot.

Supplementary Figure 5

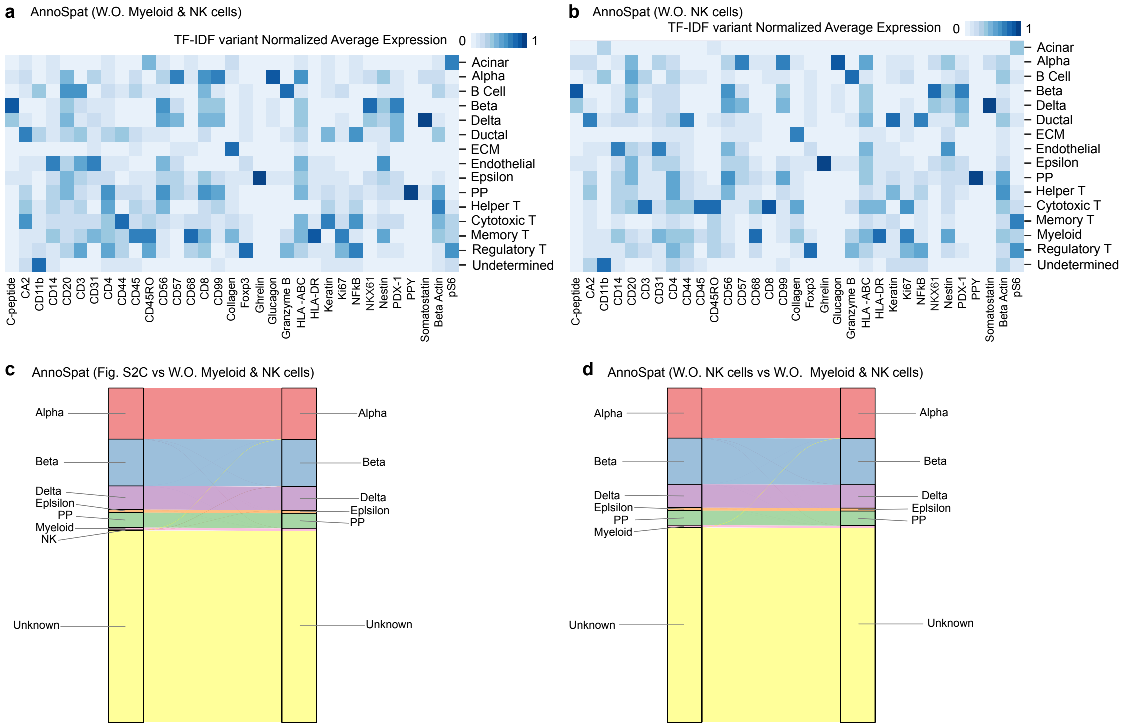

Supplementary Figure 5: Impact of Marker Protein file initialization on AnnoSpat performance. **a, b** Heatmaps showing normalized average expression of all the 33 HPAP IMC-measured proteins across AnnoSpat-annotated cell types from combined T1D and control pancreas IMC data ($n =$ 1,170,001 measured cells) when myeloid and NK cell types are removed from the Marker Protein file (a), when NK cell type is removed from the Marker Protein file (b). **c, d** Sankey plots comparing AnnoSpat-predicted cell labels with full Marker Protein file versus Marker Protein file without Myeloid and NK (c), and Marker Protein file without NK versus Marker Protein file without Myeloid and NK (d).

Supplementary Figure 6

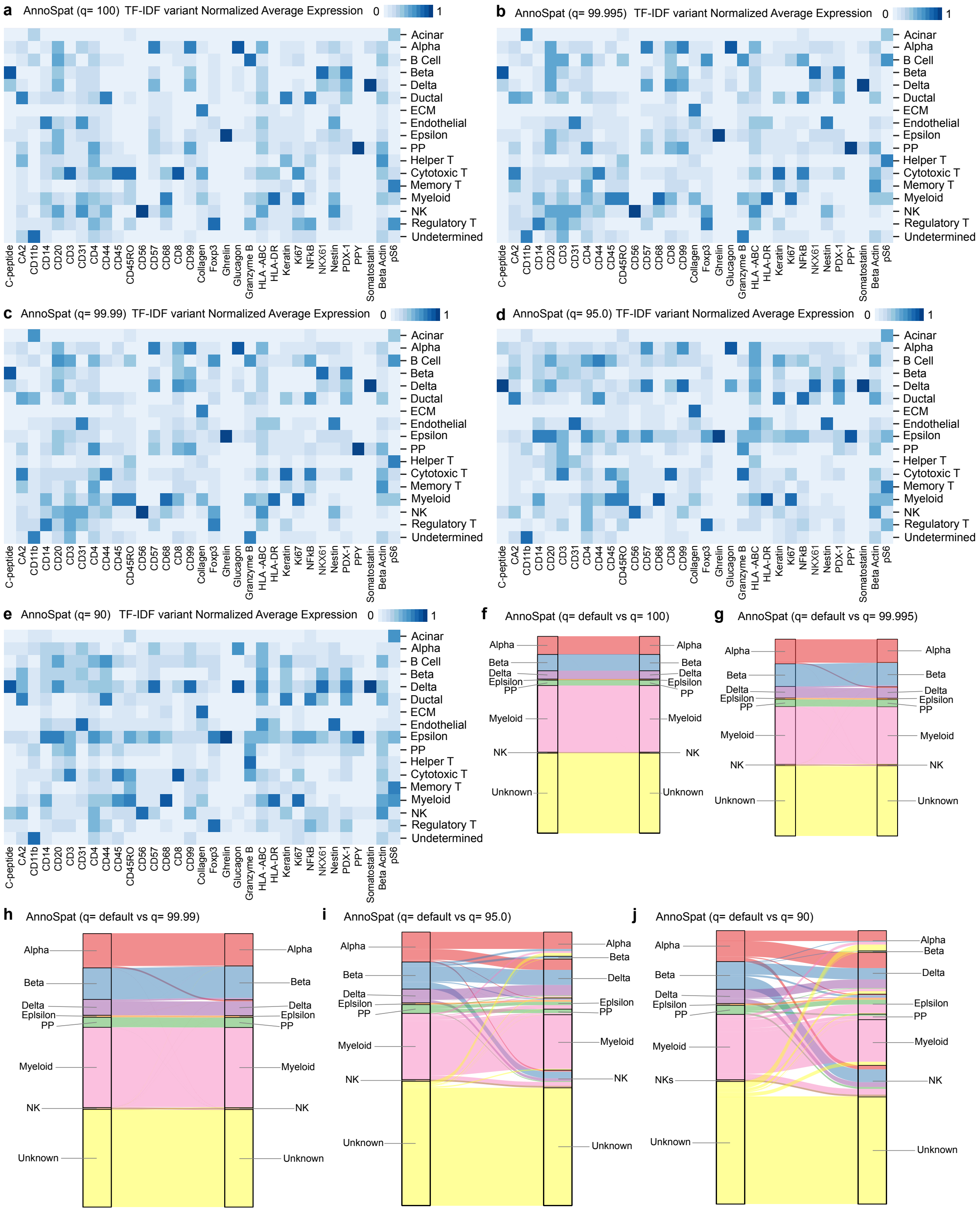

Supplementary Figure 6: Impact of cluster centroid initialization on AnnoSpat performance. **a-e** Heatmaps showing normalized average expression of all the 33 HPAP IMC-measured proteins across AnnoSpat-annotated cell types from combined T1D and control pancreas IMC data ($n =$ 1,170,001 measured cells) when cluster centroid initialization parameter q_{high} is varied. **f-j** Sankey plots comparing AnnoSpat-predicted cell labels in Figure 2c versus Figures 6a-e.

Supplementary Figure 7

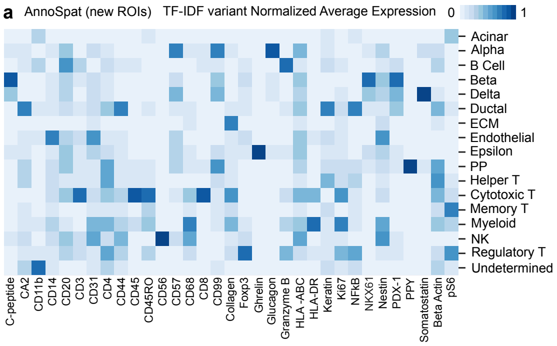

Supplementary Figure 7: AnnoSpat trained model accurately annotates cells in new ROIs. **a** Heatmap showing normalized average expression of all the 33 HPAP IMC-measured proteins across AnnoSpat-annotated cell types from combined T1D and control pancreas IMC data ($n =$ 1,170,001 measured cells) when cell types in two new ROIs were predicted by AnnoSpat pancreas trained model .

Supplementary Figure 8

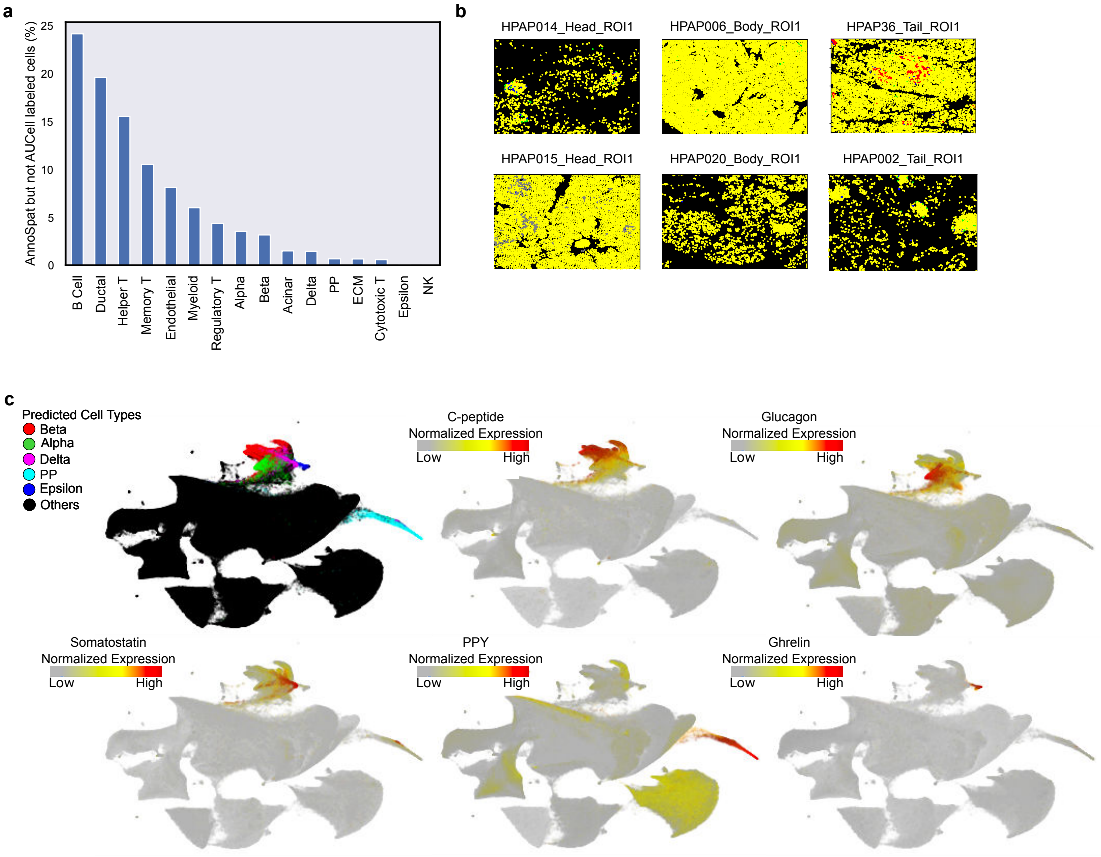

Supplementary Figure 8: Comparison of AnnoSpat and AUCell cell-type annotation. **a** Bar plots showing percentage of AnnoSpat-annotated cell types that AUCell failed to annotate. **b** Yellow pseudo-color marking AnnoSpat-annotated cells that AUCell failed to annotate on randomly selected IMC images. Other cell types are colored as before (e.g. refer to Figure 4c). **c** Comparison of normalized protein expression levels and AnnoSpat annotations across pancreatic endocrine cell types. From left to right, top to bottom: UMAP plots overlaid by AnnoSpat-predicted cell types, and normalized expression levels of c-peptide, glucagon, somatostatin, pancreatic polypeptide protein (PPY), and ghrelin in $n =$ 65,643 cells across $m =$ 141 slides of 16 pancreas donors.

Supplementary Figure 9

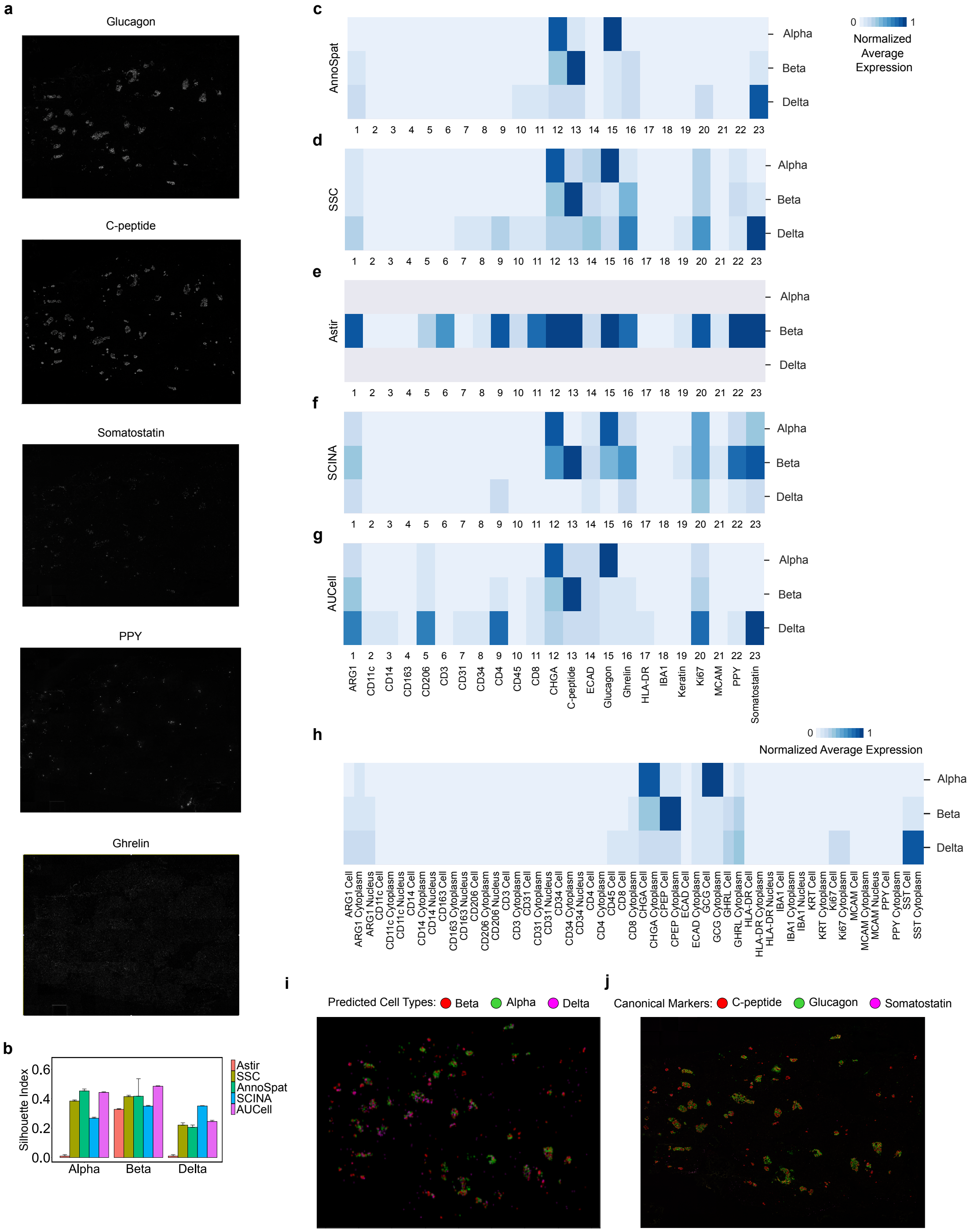

Supplementary Figure 9: Comparative analysis of AnnoSpat cell-type annotation from CODEX data. **a** From top to bottom: raw images of glucagon, c-peptide, somatostatin, PPY, and ghrelin showing non-specificity of PP and epsilon markers in CODEX experiments. **b** Bar plots with error bars showing average and standard deviation Silhouette Index (SI) for cells annotated as alpha, beta, and delta by AnnoSpat, semi-supervised clustering (SSC), Astir, SCINA, and AUCell from non-diabetic pancreas CODEX data ($m =$ 20 sets of n = 50,000 cells randomly selected from $n =$ 220,155 measured cells). **c-g** Heatmaps showing normalized average expression of all the 23 CODEX-measured proteins for cells labeled as alpha, beta, and delta by AnnoSpat, SSC, Astir, SCINA, and AUCell from non-diabetic pancreas CODEX data ($n =$ 220,155 measured cells). **h** Heatmap showing nuclear and cytoplasmic normalized average expression of all the 23 CODEX-measured proteins for cells labeled as alpha, beta, and delta by AnnoSpat from non-diabetic pancreas CODEX data ($n =$ 220,155 measured cells). **i, j** CODEX image is overlaid by AnnoSpat predicted beta, alpha, and delta labels (i) or c-peptide (beta), glucagon (alpha), and somatostatin (delta) staining (j).

Supplementary Figure 10

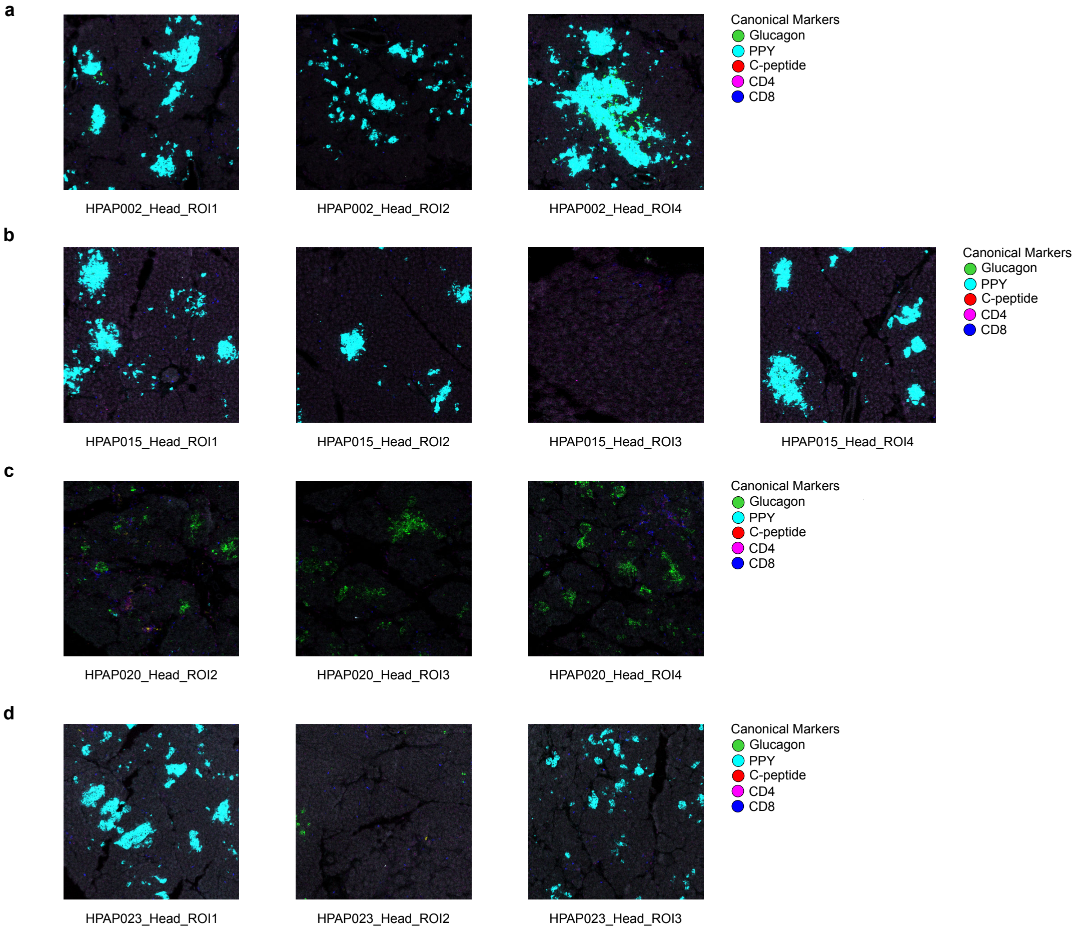

Supplementary Figure 10: PP cell count increases in the pancreas head during T1D progression. **a-d** IMC images from pancreatic head ROIs overlaid with expression levels of canonical protein markers of alpha (glucagon), beta (c-peptide), PP (PPY), helper T (CD4), and cytotoxic T (CD8) cells.

Supplementary Figure 11

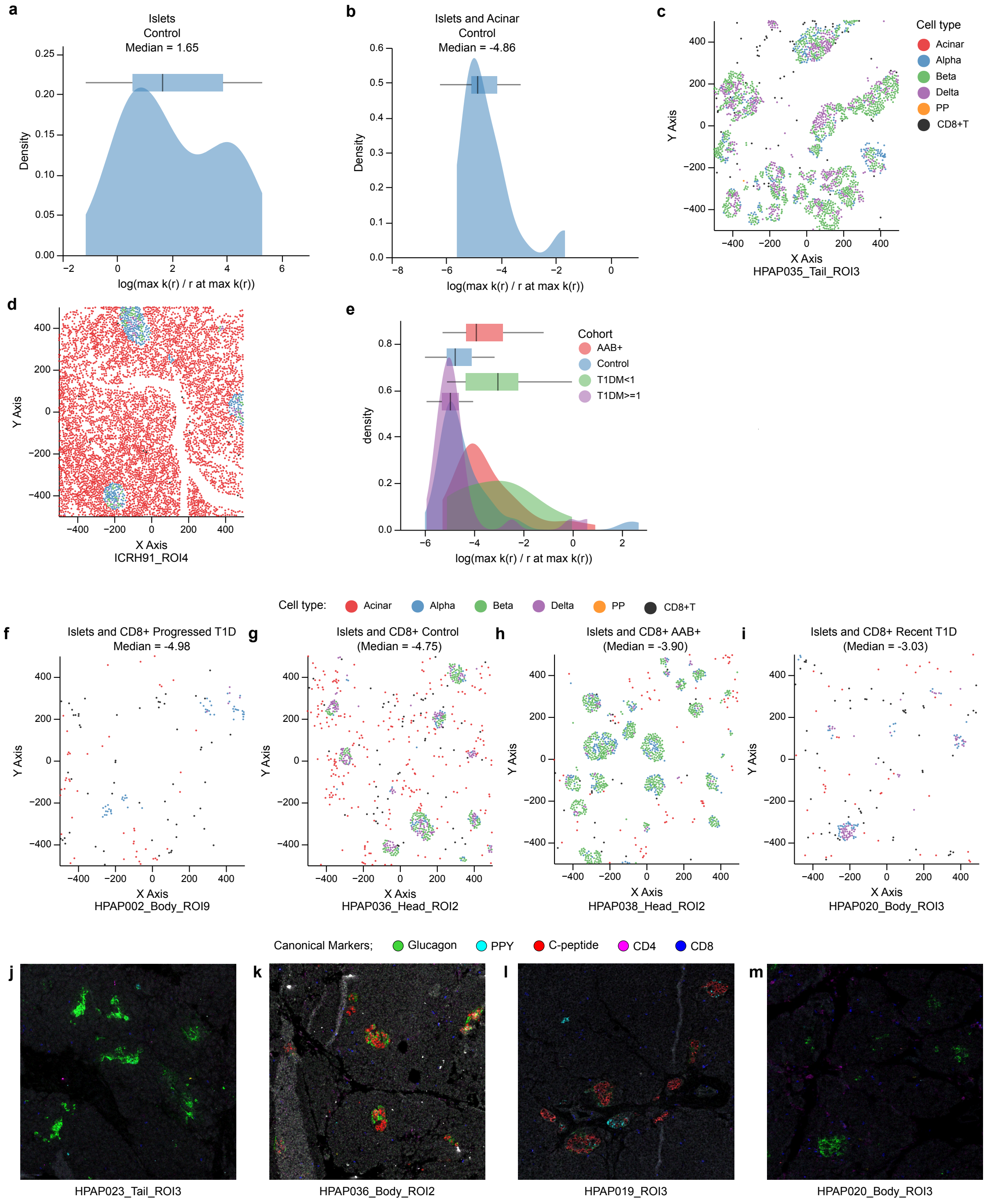

Supplementary Figure 11: The extent of $CD8^+$ T cell infiltration in islets changes during T1D progression. Analysis corresponding to Figure 6 but with an alternative summarization measure of mark cross-correlation function, which takes into account correlation value as well as distance (r): $\omega(r) = \log \frac{\max_r k_{mm}(r)}{\arg\max_r k_{mm}(r)}$. **a, b** Distributions with box-and-whisker plot overlays of $\omega(r)$ across all ROIs for endocrine cells with respect to themselves (a) or with respect to acinar cells (b). **c, d** Scatter plots showing location of cells within ROIs at the median of (a) and (b) distributions are plotted in (c) and (d), respectively. Cells are colored by AnnoSpat-predicted cell types. Endocrine cells tend to aggregate around themselves more often than with acinar cells. **e** The distributions with box-and-whisker plot overlays of $\omega(r)$ across control ($n = 48$), AAb^+ ($n = 49$), recent T1D ($n = 11$), and prolonged T1D ($n = 35$). AAb^+ and recent T1D tend to have greater aggregation of islets with $CD8^+$ T cells than control and prolonged T1D cohorts (Kruskal-Wallis Control vs T1DM ≥ 1: $p = 0.022$, AAb^+ vs T1DM < 1: $p = 0.27$). **f-i** Scatter plots showing location of cells within ROIs at the median of each cohort in (e). From lowest to highest aggregation: prolonged T1D (f), control (g), AAb^+ (h), and recent T1D (i). Cells are colored by AnnoSpat-predicted cell types. **j-m** IMC images from pancreatic ROIs overlaid with expression levels of canonical protein markers of alpha (glucagon), beta (c-peptide), PP (PPY), helper T (CD4), and cytotoxic T (CD8) cells confirming changes in the $CD8^+$ T cell infiltration in islets during T1D progression. Images in (j) to (m) correspond to scatter plots in Figures 7f to 7i, respectively. Box-and-whisker plots: center line, median; box limits, upper (75th) and lower (25th) percentiles; whiskers, $1.5 \cdot$ interquartile range; points, outliers.
