# Supplementary material for: AnnoSpat annotates cell types and quantifies cellular arrangements from spatial proteomics
Source: Nat Commun. 2024 May 3;15:3744. doi: 10.1038/s41467-024-47334-0 (PMC11068798; doi:10.1038/s41467-024-47334-0)
Supplement: Supplementary file 2 — Reporting Summary [file 41467_2024_47334_MOESM2_ESM.pdf]

nature portfolio

Corresponding author(s): Robert B. Faryabi, Gregory W. Schwartz

Last updated by author(s): 02/19/2024

Reporting Summary

Nature Portfolio wishes to improve the reproducibility of the work that we publish. This form provides structure for consistency and transparency in reporting. For further information on Nature Portfolio policies, see our Editorial Policies and the Editorial Policy Checklist.

Statistics

For all statistical analyses, confirm that the following items are present in the figure legend, table legend, main text, or Methods section.

n/a	Confirmed	
☐	☒	The exact sample size (*n*) for each experimental group/condition, given as a discrete number and unit of measurement
☐	☒	A statement on whether measurements were taken from distinct samples or whether the same sample was measured repeatedly
☐	☒	The statistical test(s) used AND whether they are one- or two-sided<br>*Only common tests should be described solely by name; describe more complex techniques in the Methods section.*
☐	☒	A description of all covariates tested
☐	☒	A description of any assumptions or corrections, such as tests of normality and adjustment for multiple comparisons
☐	☒	A full description of the statistical parameters including central tendency (e.g. means) or other basic estimates (e.g. regression coefficient) AND variation (e.g. standard deviation) or associated estimates of uncertainty (e.g. confidence intervals)
☐	☒	For null hypothesis testing, the test statistic (e.g. *F*, *t*, *r*) with confidence intervals, effect sizes, degrees of freedom and *P* value noted<br>*Give P values as exact values whenever suitable.*
☒	☐	For Bayesian analysis, information on the choice of priors and Markov chain Monte Carlo settings
☒	☐	For hierarchical and complex designs, identification of the appropriate level for tests and full reporting of outcomes
☒	☐	Estimates of effect sizes (e.g. Cohen's *d*, Pearson's *r*), indicating how they were calculated

Our web collection on statistics for biologists contains articles on many of the points above.

Software and code

Policy information about availability of computer code

Data collection	No new data was collected for this study.
Data analysis	AnnoSpat is available at https://github.com/faryabiLab/AnnoSpat<br>AnnoSpat Spatial Spatial Pattern Finder is available at https://github.com/faryabiLab/too-many-cells/, as well as https://github.com/GregorySchwartz/too-many-cells<br><br>IMC and CODEX Data: No new data has been generated in this study. IMC data were obtained from Formalin- Fixed Paraffin-Embedded (FFPE) pancreatic tissues collected by the Human Pancreas Analysis Program (HPAP) consortium (RRID:SCR_01620) as described previously (19). CODEX data were obtained from the same source. Both IMC and CODEX datasets analyzed in this study have been deposited in PANC-DB https://hpap.pmacs.upenn.edu/, data portal developed by the Faryabi Lab for HPAP, and is publicly accessible without any restriction. Relevant clinical data is available both through PANC-DB (https://hpap.pmacs. upenn.edu/) and Supplementary Data 2. In IMC, cell segmentation of all images was performed with the Vis software package (Visiopharm). All image channels were pre-processed with a 3 × 3-pixel median filter. Afterwards, cells were segmented by applying a polynomial local linear parameter-based blob filter to the Iridium-193 DNA channel of each image to select objects representing individual nuclei. Identified nuclear objects were restricted to those greater than 10 µm (36). The detected objects were dilated up to seven pixels to approximate cell boundaries. For all proteins, the average pixel intensity of the channel per cell was exported from Visiopharm and used for AnnoSpat's input. Cell locations on each ROI were also exported for AnnoSpat's input.<br><br>AnnoSpat overview: AnnoSpat is a tool to annotate single cells from their proteomic profiles and measure spatial cellular relationships using their in situ coordinates within the ROI. AnnoSpat takes as input a single-cell raw proteomic data with associated spatial information as well as a Marker Protein file listing potentially both positive and negative protein signatures associated with desired cell types. The format of the

Marker Protein file can be found in Supplementary Data 3 and Supplementary Data 10.

AnnoSpat first normalizes the protein channel intensity data to reduce the effect of outliers and varied protein intensity scales (Methods: Data Processing). AnnoSpat then randomly splits the normalized data into two partitions (training and testing sets). Cells from 50% of all ROIs are placed in the training set, while the remaining are used as the testing set. If the ROIs' disease condition/status is available, AnnoSpat can stratify the ROI split by disease status to ensure that an equal percentage of each disease status is included in each of the training and test sets.

AnnoSpat can use the cell-type labels and cellular coordinates to quantify spatial relationships between each pair of cell types (Methods: AnnoSpat's Spatial Pattern Finder). Briefly, AnnoSpats uses point process theory to quantify relationships (aggregation or repulsion) between any two cell types across a range of distances. This information is summarized with a variety of different metrics including the distance at the maximum correlation, the distance at which the correlation first becomes positive or negative, and more in order to quantify proximity relationships across ROIs. Interactive plots of each cell location with observed feature (protein expression) distributions are also outputted to facilitate data exploration (For example, see Figure 7 and Supplementary Figure 11).

AnnoSpat implements constrained K-means semi-supervised clustering (37) to identify groups of cells in the training set that are similar in proteomic space. AnnoSpat's constrained K-means clustering is initialized by "initial cluster centroids", providing cell-type aware clustering (Methods: Generation of Initial Cluster Centroids). The number of clusters is deterministic and is equal to K + 1, where K denotes the number of expected cell types in the sample. The additional (K + 1)th cluster accounts for other cell types in the experiment that are not specified in the Marker Protein file, including Unknown ones. The output of constrained K-means produces the cells that are predicted to be related and thus are used by AnnoSpat as a training set to learn the label of the remaining cells by training an extreme learning machine classifier (ELM) (Methods: Training Extreme Learning Machine Classifier) (12). The trained model is saved to label cells from other data sources, eliminating the need for re-clustering or re-training whenever new data is available.

AnnoSpat data processing
To reduce the effect of outliers, AnnoSpat first calculates Data matrix D by log transforming cell-by-protein channel intensity (expression) after addition of pseudo-count 1. Specifically, $d_{c \times p} = e_{c \times p} + 1 $, where $e_{c \times p}$ is the expression of protein $p $ channel in cell c. Then, AnnoSpat unit normalizes the log-transformed intensity matrix to scale each cell vector to unit length. This projects each cell to a unit sphere in the proteomic space. We denote the normalized proteomic matrix by X obtained from scaling each row d_i of D as follows:

\begin{equation}
    x_{i} = \frac{d_i}{||d||} \text{ , where} ||d_i|| = (\sum\limits_{j=1}^{P} ||d_j||^2)^{1/2},
\end{equation}

where, $||d_i||$ denotes the l_2 or Euclidean norm of i th cell. P is the number of measured proteins.

This step accounts for variable expression across proteins and correlates the Euclidean distances (used for clustering) between cell vectors and cosine distances in the proteomic space. Compared to euclidean distance, the angle between the cell vectors in proteomic space better reflects cell-cell similarities/differences (38).

Generation of initial cluster centroids: As opposed to traditional K-means where the initial cluster centroids are randomly selected, AnnoSpat implements constrained K-means that follows a more cell-type aware approach (37). Initial cluster centroids are obtained from representatives of each cluster (cell-type here). AnnoSpat calculates initial cluster centroids by taking the mean of representative cells R_k for each cluster $k = 1, \ldots, K+1$. The number of clusters is one more than the number of cell types K; an extra (Unknown) cluster accounts for cell types not included in the Marker Protein file.

AnnoSpat obtains the cluster representations $R_1, R_2, \ldots, R_{K}$ by:

1- Obtaining positive and negative markers M^+ and M^- from the Marker Protein file.

2- Calculating the score M_c for c^{th} cell type by multiplying the protein intensities corresponding to positive markers and the compliment of protein intensities corresponding to negative markers as follows:
\begin{equation}
   M_c = \prod \limits_{ i \in M+}{X_i } * \prod\limits_{ j \in M-}{ (max(X_j) - X_j) }, \quad c=1, \ldots, K.
\end{equation}

3- Selecting cell representatives $R_1, R_2, \ldots, R_{K}$ of cell types $c=1, \ldots, K$ in the Marker Protein file such that they have
\begin{equation}
   M_c > M_{c,high} \quad \text{ and } \quad M_c < M_{c,max}, \text{ where}
 \end{equation}

[\
   M_{c,high} = \percentile (M_c, q_{high}) \quad \text{and} \quad M_{c,max} = \percentile(M_c, q_{max}).
\]

The value q_{high} is adaptive and can be optionally chosen based on prior knowledge of the number of cells from the cell type present in the data (defaulting to the 95th percentile). Here, q_{high} was set to $ 99 \leq q_{high} \leq 99.9$ and $ 99.5 \leq q_{high} \leq 99.99 $ for various cell types in the analysis of pancreas IMC and CODEX data, respectively. $M_{c,high}$ is the score cut-off to pick cluster representative cells as the ones having a very high score M_c corresponding to the cth cell type. The threshold q_{max} is set to 100 or a value slightly less than that to make sure that assay artifacts are not included in the initial cluster centroid calculation. Here, q_{max} was set to 99.999 and 100 for the analysis of pancreas IMC and CODEX data, respectively.

M^+ and M^- cannot be defined for the Unknown cluster, since multiple cell types could be captured in this cluster. Hence, after calculation of M_c for cell types $c=1, \ldots, K$ with specified markers, the intersection of all cells with low M_c value for all cell types $c=1, \ldots, K$ are identified. The intuition here is that cell types whose markers are not specified in the Marker Protein file (i.e. Unknown cells) should not be representatives of any cell type $c=1, \ldots, K$, and thus M_c score should be low for the Unknown cells with respect to all the cell types $c=1, \ldots, K$ with known markers.

In other words:

1- For each cell type $c=1, \ldots, K$,
  a- Calculate M_c (using M^+ and M^- of cell types defined in the Marker Protein file)
  b- Obtain the cells with $M_c < M_{c,low}$ where,
  \begin{equation}
  M_{c,low} = \percentile(M_c, q_{low}).
  \end{equation}
  The threshold q_{low} defines the cut-off to chose cells with expression $< M_{c,low}$ in cell types c.
  This will pick cells U_c that belong to cell type c with very low probability.
2- The Unknown class is identified by taking the intersection of $U_{1}, \ldots, U_{K}$ sets. These cells are least likely to represent any of cell types defined in the Marker Protein file.

AnnoSpat performs the above procedure to assign cluster representative cells in decreasing order of cell-type abundance (representative of more abundant cell types are selected first). Cell-type abundance acts as a proxy for the expected number of cells for each cell type and is obtained by summing cell intensities of the scale-normalized canonical protein markers.

Once the cell representatives $R_1, R_2, \ldots, R_{K+1}$ have been assigned, AnnoSpat computes initial centroids $\overline x_{k}$ for cluster $k = 1, \ldots, {K+1}$ by taking the average across the representative cells R_{k} as follows:

\begin{equation}
\overline x_{kj}= \frac{1}{|R_k|}\sum\limits_{x_i \in R_k} {x_{ij}} \text{ , for } j = 1, \ldots, P
\end{equation}

where x_{ij} represents the intensity of the jth protein in i^{th} cell.

Cell labeling with semi-supervised clustering: AnnoSpat takes the cell representatives R_k's and initial cluster centroids $\overline x_{k}$'s and iteratively runs constrained K-means algorithm on the cells from 50\% of the ROIs included in the training set as shown in Algorithm 1. L_i denotes the cluster label assigned to the ith cell and C_k denotes the set of cells in cluster k. The assigned cell labels are the predicted cell types of training data for the AnnoSpat's Annotator.

Algorithm 1: Constrained K-means
 \textbf{Initialize} K, n
 \textbf{Input}: Normalized data X, initial centroids \{$\overline x_{1}, \ldots, \overline x_{K+1}$\} and cell representative\{$R_1,R_2.....R_{K+1}$\}
 \textbf{For} $iter=1,2,\ldots,n$
   Cluster assignment:
     When $x_i \in R_k$, $L_i = k$
     Otherwise, $L_i = \operatorname{argmin}_{k} ||x_{i}-\overline x_{k}||^2$
   Centroid computation:
     $\overline x_{k}= \frac{1}{|C_k|}\sum\limits_{x_i \in C_k} {x_{i}}$ \\
 \textbf{End For}
 \textbf{Return}: Labels L, Centroids $\overline x$ \\

Training Extreme Learning Machine classifier: AnnoSpat uses the cell-type labels L predicted by its semi-supervised clustering algorithm as training labels Y_{TR} to then learn an ELM classifier (12). The classifier predicts the label of remaining cells in new ROIs not included in the training data. We implemented ELM in AnnoSpat because it is a single-layer feed-forward neural network classifier and does not need to be iteratively tuned via backpropagation. This would enable AnnoSpat to learn accurate cell type prediction models markedly faster than gradient-based learning techniques.

Comparative analysis during the design of AnnoSpat confirmed earlier studies (12) and showed that, while ELM and Support Vector Machines (SVM) provide comparable accuracy in annotating $\sim$1,170,000 cells in our IMC dataset (Figures \ref{figS2}c and \ref{figS2}h), ELM was $\sim$2 times faster than SVM (73.9 vs 159.9 sec). This characteristics makes it a more suitable classifier for near-online annotation of atlas scale data sets, including HPAP.

AnnoSpat's ELM is implemented as follows:

 1- Assign input layer weights W_{I} and bias b_{I} randomly from normal distributions:
 \begin{equation}
 W_{I} \sim \mathbb{N}(0,\mathbb{I})
 \end{equation}
 \begin{equation}
 b_{I} \sim [{N}(0,1)]

\end{equation}

2- Compute hidden layer output H:
\begin{equation}
H = \phi (W_{I}*X_{TR} +b_{I})
\end{equation}
Here, ϕ denotes the activation function used at the hidden layer, and X_{TR} is the normalized protein intensity of training set.

3- Compute the output layer weights W_{O}
\begin{equation}
W_{O} = H^{\dagger} *Y_{TR}
\end{equation}
Here $H^{\dagger}$ is the Moore–Penrose inverse of hidden layer output matrix H. The training labels Y_{TR} are transformed into a one-hot encoded format to avoid ordinal relationship interpretability between cell types by the model.

Once the output weights are learned, the types (labels) of new cells Y_{TS} can be predicted from their normalized protein expression X_{TS} by the learned weights in ELM:

\begin{equation}
Y_{TS} = \phi(W_{I} *X_{TS} +b_{I})*W_{O}
\end{equation}

To demonstrate AnnoSpat's generalizability, we additionally used the trained AnnoSpat model to annotate cells in two additional ROIs from HPAP organ donors that were not part of the original 143 slides of 19 donors, and examined the accuracy of cell-type annotation.

Cell type prediction with unsupervised clustering algorithms: To predict cell types with unsupervised clustering, we used K-means, Seurat (16), FlowSOM (18) and PhenoGraph (17) clustering followed by differential protein expression analysis between cells in each cluster versus cells in all other clusters.

K-means clustering was implemented using scikit-learn library with default parameters. Similar to AnnoSpat, the number of clusters for K-means clustering n clusters was set to 17.

CreateSeuratObject function from Seurat R package was used to process the protein expression table. NormalizeData and ScaleData functions were then used to for data normalized and scaled. RunPCA function was used to perform principal component analysis on the normalized counts. Given the number of cell types that could be detected by the HPAP IMC panel, we used FindNeighbors function with dims = 1:30, and FindClusters function with resolution equal to 0.3 (T1D), 0.2 (Control), and 0.2 (Combined), which resulted in 17, 19, and 22 clusters in T1D, Control and Combined cohorts, respectively.

To cluster cells with FlowSOM, first, read.flowSet function from openCyto R package was used to convert CSV files of raw protein expression values to FCS objects (note: this function is not exposed in the package's API). Then, FlowSOM, BuildSOM, and BuiltMST functions from FlowSOM R package were used to construct self-organizing map (SOM) and minimum spanning tree (MST). Given the number of cell types that could be detected by the HPAP IMC panel, we finally used metaClustering consensus function with k) equal to 17 to perform Meta-clustering on MST.

Phenograph expects normalized expression values. Hence, NormalizeData function from Seurat R package was used to normalize raw expression values. pandas.read csv method from Pandas dataframe was used to input normalized protein expression CSV files. Given the number of cell types that could be detected by the HPAP IMC panel, we used phenograph.cluster function with k equal to 1000 (T1D), 500 (Control), and 1000 (Combined), which resulted in 21, 23, and 37 clusters in T1D, Control and Combined cohorts, respectively.
The out of each clustering method was used for a series of one-vs-all-others differential protein expression analyses. We used Mann-Whitney U test to determine the significance of differences in protein expression levels for cells in each cluster versus cells in all other clusters. The log2 fold change (FC) of each cluster's mean expression of a given protein was used as a further measure of difference between cell clusters. The final measure of significance for each cluster was calculated as −log10(p−value)×log2 FC. To assign cell type labels. the most deferentially expressed protein for each cluster was queried against the Marker Protein file in Supplementary Data 3 for cell type assignment. For all clustering algorithms, the order of cell type label assignment was same as the order of columns in Supplementary Data 3.

Comparison of algorithm-annotated and expert-annotated cell types: Endocrine composition of expert-annotated cell types in different sections of pancreata from five HPAP donors were obtained from (19). Wang et al. borrowed approaches commonly used in flow cytometry data analysis to manually annotate the cells in each individual ROI (19). In summary, the cells were annotated by a combination of manual gating and thresholding based on Gaussian mixture models that were used to separately identify positive and negative cutoffs for each individual protein channel in each ROI, followed by so-called Boolean rules. These rules start assigning the cells to alpha, beta, delta, PP, epsilon, followed by other cell types with available markers in HPAP IMC assay, in that order, based on "Positive" and "Exclusion" markers as listed in (19). For each (donor, section), we used minimum Kullback-Leibler (KL) divergence to determine distance between the Wang et al. reported endocrine cell type distribution and AnnoSpat-, SSC-, Astir-, SCINA-, or AUCell-annotated endocrine cell type distribution. Two endocrine cell type distributions were called discordant if their minimum KL divergence was greater than 0.4.

Data processing for visualization: Data for Heatmaps in Figure 2, as well as Supplementary Figures 2c-g, 3, 4a-c, 5a-b, 6a-e and 7 have been normalized to penalize the expression of non-specific proteins using an analog variant of TF-IDF (term frequency-inverse document frequency) normalization after min-max scaling of protein expression. The specificity of a protein can be quantified as an inverse function of the number of cell types in which it is expressed (its abundance across various cell types). Hence, the normalized value of protein p_i is obtained by multiplying each value by the logarithm of ratio of total protein abundance p_{total} in the data and the abundance of that protein across all cell types p_{sum}. If p is the expression of protein p_i in cell c_j, then the normalized value is calculated by:

\begin{equation}
p^{TF,IDF}_{i,j}= p*log(\frac{p_{total} }{p_{sum}}).
\end{equation}

In min-max normalization, min and max values are the 0.01th and 99.99th percentile expression, respectively.

AnnoSpat's Spatial Pattern Finder: quantification of cell proximity pattern

In order to quantify the relationships between cell types in the T1D pancreas, we interpreted the cell locations and cell type labels as a marked point pattern. A point pattern provides the locations of observations; here, cell locations are represented as Cartesian coordinates. Each cell can have additional features known as marks; here, each cell's mark is the predicted cell type. By realizing the marked point pattern as a random marked point process, we can quantify cell type spatial relationships. A point process is a random set of points, where the number of points and their locations are both random. Using point process theory, we can understand the relationship between cell types not as a single index, but rather as many values resulting in formulation of a given function of distance \(r\).

The standard model of a point process \(\mathbb{\Psi}\) assumes that the process extends all space, but the observed region is bounded by a window \(W\). Then we can define the data as an unordered set (39)

\begin{equation}
\mathbf{\psi} = {\psi_1, \dots, \psi_n}, \psi_i \in W, n > 0,
\end{equation}
\noindent the point pattern of \(\mathbf{\Psi}\).

Now we can define our ROI within the context of marks. Consider the marked point pattern as an unordered set of cells observed within a window \(W\) with marks in \(M\),

\begin{equation}
\mathbf{\gamma} = {(\psi_1, m_1), \dots, (\psi_n, m_n)}, \psi_i \in W, m_i \in M,
\end{equation}

where \(\psi_i\) is the location and \(m_i\) is the mark of cell \(i\), respectively (39). Marks may be continuous real numbers, such as cell size, or discrete, such as cell type. Our objective is to quantify the dependence between the marks of two cells of distance \(r\) apart in the marked point process \(\mathbf{\Gamma}\). This dependence, known as the mark correlation function \(k_f(r)\), is informally defined as (39, 40)

\begin{equation}
k_f(r) = \frac{\mathbb{E}_{i,j}[f(M_i, M_j)]}{\mathbb{E}[f(M,M')]},
\end{equation}

where \(M_i,M_j\) are marks of two cells separated by distance \(r\), \(M,M'\) are independent realizations of the marginal distribution of marks, and \(\mathbb{E}\) is the intensity of a point process, or the average density of points (the expected number of points per unit area), and where \(\mathbb{E}_{i,j}\) is the conditional expectation that there exist cells at locations \(i\) and \(j\) separated by distance \(r\). While \(f\) is any function that returns a non-negative real value, we commonly use \(f(m_1, m_2) = m_1m_2\) for continuous marks and \(f (m_1,m_2) = \mathbb{1}(m_1,m2) = 1\) where \(m_1 = m_2\) and \(= 0\) for everything else for discrete (categorical) marks (39). Then, \(k_f (r) = 1\) suggests a lack of correlation such that under random mark labeling, \(k_f(r) \equiv 1\). The interpretation of greater than or less than 1 would be determined by the chosen function \(f\), but throughout this study we interpret \(> 1\) as correlated and \(< 1\) as anti-correlated. This mark correlation function, however, assumes that cell type would be a single mark and does not specify the relationship between, for instance, CD8^{+} T cells and islet cells.

To understand the relationship between any two cell types, we expand the mark correlation function \(k_f(r)\) to define the mark cross-correlation function, \(k_{mm}(r)\). Here, instead of \(m_i \in M\) as a single mark, we define \(\mathbb{m}_{ia} \in M\) as the value of mark \(a\) in cell \(i\) from the row vector of marks \(\mathbb{m}_i\) attached to cell \(i\). Instead of a single mark for cell type, we convert the mark into a mark row vector \(m_i\) for cell \(i\) containing \(c\) entries, where each index \(0 < j \le c\) represents an indicator value for cell type \(a\). In short, \(\mathbb{m}_{ia} = 1\) indicates that the cell \(i\) is of cell type \(a\).

Using this expanded mark vector, we can define the mark cross-correlation function (39) as

\begin{equation}
k_{mm}(r) = \frac{\mathbb{E}_{i,j}[f(M_{ia}M_{jb})]}{\mathbb{E}[f(M_a,M_b)]},
\end{equation}

where \(M_{ia}\) and \(M_{jb}\) are the marks \(a\) and \(b\) attached to cells \(i\) and \(j\), respectively, while \(M_a\) and \(M_b\) are independent random values drawn from all cells at mark indices \(a\) and \(b\), respectively. Here, \(f\) is defined as with the mark correlation function. Using categorical marks for cell types, we then interpret \(k_{mm}(r) > 1\) as correlated, \(< 1\) as anti-correlated, and \(= 1\) as random. We carried out all mark cross-correlation analyses using the spatstat R package (39).

The output of each mark cross-correlation function on an ROI is a series of correlation values as a function of distance \(r\). To compare across several ROIs, we summarized each curve by either the \(r\) at the maximum \(k_{mm}(r)\) (\(\max_r{k_{mm}(r)}\)) (Figure 7) or the log-transformed ratio of the maximum \(k_{mm}(r)\) to the \(r\) at the maximum \(k_{mm}(r)\) (\(\log{\frac{\max_{r}{k_{mm}(r)}}{\argmax_{r}{k_{mm}(r)}}}\)) (Figure S11). The former value decreases with increasing aggregation (the highest correlation is with cells with smaller \(r\)) while the latter increases with increasing aggregation. To compare distributions, we used Kruskal-Wallis one-way analysis of variance for multiple hypotheses followed by pairwise Mann-Whitney \(U\) tests.

For manuscripts utilizing custom algorithms or software that are central to the research but not yet described in published literature, software must be made available to editors and reviewers. We strongly encourage code deposition in a community repository (e.g. GitHub). See the Nature Portfolio guidelines for submitting code & software for further information.

Data

Policy information about availability of data

All manuscripts must include a data availability statement. This statement should provide the following information, where applicable:

- Accession codes, unique identifiers, or web links for publicly available datasets
- A description of any restrictions on data availability
- For clinical datasets or third party data, please ensure that the statement adheres to our policy

No new data has been generated in this study. The IMC and CODEX datasets analyzed in this study have been deposited in PANC-DB (https://hpap.pmacs.upenn.edu/), the data portal of Human Pancreas Analysis Program (HPAP) consortium (RRID: SCR_01620) developed by the Faryabi Lab, and is publicly accessible without any restriction. Relevant clinical data is also available both through PANC-DB (https://hpap.pmacs.upenn.edu/) and Supplementary Data 2.

Research involving human participants, their data, or biological material

Policy information about studies with human participants or human data. See also policy information about sex, gender (identity/presentation), and sexual orientation and race, ethnicity and racism.

Reporting on sex and gender	N/A
Reporting on race, ethnicity, or other socially relevant groupings	N/A
Population characteristics	N/A
Recruitment	N/A
Ethics oversight	N/A

Note that full information on the approval of the study protocol must also be provided in the manuscript.

Field-specific reporting

Please select the one below that is the best fit for your research. If you are not sure, read the appropriate sections before making your selection.

- [x] Life sciences
- [ ] Behavioural & social sciences
- [ ] Ecological, evolutionary & environmental sciences

For a reference copy of the document with all sections, see nature.com/documents/nr-reporting-summary-flat.pdf

Life sciences study design

All studies must disclose on these points even when the disclosure is negative.

Sample size	No sample size calculation was performed. Data presented in this manuscript is part of HPAP project, which is an ongoing NIH-funded project to characterize human pancreata from healthy and T1D donors. We analyzed the entire available HPAP IMC dataset at the time of initial submission, which included 143 images form 16 pancreatic donors. HPAP donor information is summarized in Table S2.
Data exclusions	All the high quality IMC data collected to date by the HPAP program were used in this study. No sample were excluded. However, the channels with none-specific antibodies were excluded to improve the accuracy of benchmarking analysis.
Replication	These are human donor samples on a hard to harvest (and in some instances rare) population of cells, and therefore, replication is not plausible.
Randomization	Randomization was not carried out as they were not relevant to our analysis. This paper proposes a method for analysis of IMC data does not correlate donor clinical information with phenotypic / genotypic data.
Blinding	Blinding was not carried out because the major approach used in this study, IMC, is generally considered to be unbiased.

Reporting for specific materials, systems and methods

We require information from authors about some types of materials, experimental systems and methods used in many studies. Here, indicate whether each material, system or method listed is relevant to your study. If you are not sure if a list item applies to your research, read the appropriate section before selecting a response.

Materials & experimental systems

n/a	Involved in the study	
☐	☒	Antibodies
☒	☐	Eukaryotic cell lines
☒	☐	Palaeontology and archaeology
☒	☐	Animals and other organisms
☒	☐	Clinical data
☒	☐	Dual use research of concern
☒	☐	Plants

Methods

n/a	Involved in the study	
☒	☐	ChIP-seq
☒	☐	Flow cytometry
☒	☐	MRI-based neuroimaging

Antibodies

Antibodies used

Tables S1 and S3 list the IMC and CODEX antibodies respectively.

Validation

No new data was generated for this manuscript.

Adapted from the IMC paper in which this approach was described, Y. J. Wang et al., Multiplexed In Situ Imaging Mass Cytometry Analysis of the Human Endocrine Pancreas and Immune System in Type 1 Diabetes. Cell Metab 29, 769-783 e764 (2019): "20 of the 33 antibodies used in the current panel were directly purchased from FluidigmR (https://www.fluidigm.com). For the remaining 13 antibodies, carrier-free antibodies were purchased from different vendors and tested by immunofluorescent staining on FFPE sections. Based on anticipated epitope abundance, either pancreas or spleen sections were used for antibody testing. The staining results of antibodies were assessed by board-certified pathologists. Antibodies producing the expected results were conjugated with lanthanide metals using the Maxpar X8 metal conjugation kit following manufacturer's protocol (Fluidigm 201300). Post-conjugation, antibody specificities were again tested using immunofluorescent staining, followed by titration in the IMC platform. The antibody characterization procedure and results are shown in Figures S1A and S1B and Table S1. More details on antibody quality assessment can be found in Tables S2 and S3."

Adapted from the IMC paper in which this approach was described, Y.J. Wang et al. Single-Cell Mass Cytometry Analysis of the Human Endocrine Pancreas. Cell Metab. 24(4):616-626 (2016): "Antibodies were validated by the following three methods. (1) Immunofluorescent labeling (Figures S1A–S1E): the labeling efficiency of each antibody was tested by direct labeling of human islet cells in suspension. Briefly, human pancreatic cells were dissociated and labeled with the test antibodies following the same procedure as when performing mass cytometry. Subsequently, Cy3-conjugated secondary antibodies were applied. Cells were cytospun onto microscope slides and imaged under a fluorescent scope. Only those antibodies displaying the expected staining patterns were used in downstream experiments. (2) Flow cytometry (Figure S1F): cells were dissociated and labeled as in mass cytometry experiment, followed by secondary antibody staining in the Cy3 channel. Cellular events were subsequently acquired by a BD LSRII following a standard flow cytometry protocol. (3) Stimulation followed by CyTOF2 sample acquisition (Figure S1G): human islets were (a) incubated in retinoic acid at 50 nM final concentration for 72 hr; (b) serum starved overnight, followed by stimulation with 1.25 ng/ml Leptin for 4 hr; or (c) serum starved for 48 hr, followed by stimulation with Prolactin at 200 ng/ml for 30 min. After stimulation, cells were dissociated and processed following a normal mass cytometry sample preparation protocol. Antibodies that passed initial quality control were titrated in CyTOF with 1:100, 1:200, 1:500, 1:1,000, and 1:10,000 dilutions. Unlabeled cells were used as a negative control."
