# Supplementary material for: AnnoSpat annotates cell types and quantifies cellular arrangements from spatial proteomics
Source: Nat Commun. 2024 May 3;15:3744. doi: 10.1038/s41467-024-47334-0 (PMC11068798; doi:10.1038/s41467-024-47334-0)
Supplement: Supplementary file 16 — Description of Additional Supplementary Files [file 41467_2024_47334_MOESM16_ESM.pdf]

Supplementary Data 1. IMC antibody panel.

Supplementary Data 2. HPAP pancreas donor information.

Supplementary Data 3. AnnoSpat's Marker Protein file input for annotating all the possible 16 cell types using 33 HPAP IMC-measured proteins.

Supplementary Data 4. SI scores for labeling endocrine cells from IMC samples of T1D donors' pancreata (T1D Cohort). Numbers in parentheses: standard deviation. NA: no or not enough cells were annotated.

Supplementary Data 5. SI scores for labeling endocrine cells from IMC samples of non-diabetic donors' pancreata (Control cohort). Numbers in parentheses: standard deviation. NA: no or not enough cells were annotated.

Supplementary Data 6. SI scores for labeling endocrine cells from IMC samples of combined non-diabetic and T1D donors' pancreata (Combined cohort). Numbers in parentheses: standard deviation. NA: no or not enough cells were annotated.

Supplementary Data 7. Fraction of endocrine cells labeled by each algorithm from IMC samples of T1D, non-diabetic (Control), and combined T1D and control (Combined) donors' pancreata.

Supplementary Data 8. Mean and standard deviation of run-time for listed algorithms to annotate cells from IMC samples of T1D, non-diabetic (control), and combined T1D and control (Combined) donors' pancreata. Each algorithm was run fifteen times on data sets of n = 374,397, n = 795,604, and n = 1,170,001 cells from IMC samples of T1D, control, and combined T1D and control donors using a machine with Ubuntu 20.04, 1.05TB Memory, Intel Xeon Gold CPU 6230R @ 2.1GHz, 2 physical processors 52 cores, and 104 threads.

Supplementary Data 9. CODEX antibody panel.

Supplementary Data 10. AnnoSpat's marker protein file input for annotating the listed cell types from CODEX antibodies.

Supplementary Data 11. SI scores for labeling alpha, beta, and delta cells from a non-diabetic donor pancreas CODEX. Numbers in parentheses: standard deviation. NA: no cell was annotated.

Supplementary Data 12. Fraction of expert-annotated endocrine cell types in different regions of pancreata from donors studied in (19).

Supplementary Data 13. Minimum Kullback-Leibler (KL) divergence for endocrine cell type distribution for each (donor, section) in Wang et al. (19) versus AnnoSpat, SSC, Astir, SCINA, and AUCell.
